# Impact of endorectal filling on interobserver variability of MRI based rectal primary tumor delineation

**DOI:** 10.1016/j.ctro.2022.09.002

**Published:** 2022-09-21

**Authors:** Monica Lo Russo, Marcel Nachbar, Aisling Barry, Shree Bhide, Amy Chang, William Hall, Martijn Intven, Corrie Marijnen, Femke Peters, Bruce Minsky, Paul B. Romesser, Reith Sarkar, Alex Tan, Simon Boeke, Daniel Wegener, Sarah Butzer, Jessica Boldt, Sergios Gatidis, Konstantin Nikolaou, Daniela Thorwarth, Daniel Zips, Cihan Gani

**Affiliations:** a Department of Radiation Oncology, University Hospital and Medical Faculty, Eberhard Karls University Tübingen, Germany; b Section for Biomedical Physics, Department of Radiation Oncology, University Hospital Tübingen, Germany; c Radiation Medicine Program, Princess Margaret Cancer Centre, University Health Network, Toronto, ON, Canada; d Radiotherapy and Imaging, The Royal Marsden NHS Foundation Trust and the Institute of Cancer Research, London, United Kingdom; e Hong Kong Sanatorium and Hospital, Hong Kong, China; f Department of Radiation Oncology, Medical College of Wisconsin, Milwaukee, WI, USA; g Department of Radiation Oncology, University Medical Center Utrecht, Utrecht, The Netherlands; h Department of Radiation Oncology, Netherlands Cancer Institute, Amsterdam, the Netherlands; i Department of Gastrointestinal Radiation Oncology, University of Texas MD Anderson Cancer Center, Houston, TX, USA; j Department of Radiation Oncology, Memorial Sloan Kettering Cancer Center, New York, NY, USA; k GenesisCare, Australia; l Department of Radiology, University Hospital and Medical Faculty, Eberhard Karls University Tübingen, Germany; m German Cancer Research Center (DKFZ) Heidelberg and German Consortium for Translational Cancer Research (DKTK), Partner Site Tübingen, Tübingen, Germany

**Keywords:** Rectal cancer, MR-guided radiotherapy, MRI-Linac, Inter-observer agreement

## Abstract

**Background:**

Online adaptive MR-guided radiotherapy allows for the reduction of safety margins in dose escalated treatment of rectal tumors. With the use of smaller margins, precise tumor delineation becomes more critical. In the present study we investigated the impact of rectal ultrasound gel filling on interobserver variability in delineation of primary rectal tumor volumes.

**Methods:**

Six patients with locally advanced rectal cancer were scanned on a 1.5 T MRI-Linac without (MRI_e) and with application of 100 cc of ultrasound gel transanally (MRI_f). Eight international radiation oncologists expert in the treatment of gastrointestinal cancers delineated the gross tumor volume (GTV) on both MRI scans. MRI_f scans were provided to the participating centers after MRI_e scans had been returned. Interobserver variability was analyzed by either comparing the observers’ delineations with a reference delineation (approach 1) and by building all possible pairs between observers (approach 2). Dice Similarity Index (DICE) and 95 % Hausdorff-Distance (95 %HD) were calculated.

**Results:**

Rectal ultrasound gel filling was well tolerated by all patients. Overall, interobserver agreement was superior in MRI_f scans based on median DICE (0.81 vs 0.74, p < 0.005 for approach 1 and 0.76 vs 0.64, p < 0.0001 for approach 2) and 95 %HD (6.9 mm vs 4.2 mm for approach 1, p = 0.04 and 8.9 mm vs 6.1 mm, p = 0.04 for approach 2). Delineated median tumor volumes and inter-quartile ranges were 26.99 cc [18.01–50.34 cc] in MRI_e and 44.20 [19.72–61.59 cc] in MRI_f scans respectively, p = 0.012.

**Conclusions:**

Although limited by the small number of patients, in this study the application of rectal ultrasound gel resulted in higher interobserver agreement in rectal GTV delineation. The endorectal gel filling might be a useful tool for future dose escalation strategies.

## Highlights

• Interobserver variability of MRI based primary tumor delineation studied by a panel of international experts.
• Interobserver variability significantly improves after the application of 100 cc of rectal ultrasound gel.
• The approach presented possibly improved dose escalation protocols in rectal cancer.

## Introduction

Over the last several years non-operative management of rectal cancer following a clinical complete response (cCR) to radiotherapy has become a widely accepted approach [8]. However, using conventional chemoradiotherapy regimens pathological complete response rates (pCR) are generally below 20 % [12]. Therefore, there is significant interest in optimizing neoadjuvant strategies to increase complete response rates and facilitate non-operative management for a larger fraction of rectal cancer patients. Besides intensified concomitant and/or sequential chemotherapy, hyperthermia and delayed response assessment, radiation dose escalation is a promising way to increase local response rates in rectal cancer [3], [7], [15], [8], [11]. However, rectal tumors show considerable interfractional motion resulting in large safety margins required to ensure adequate target volume coverage using a non-adaptive cone-beam computed tomography (CBCT) based approach [10]. Moreover, non-adaptive dose escalation strategies miss the opportunity to reduce radiation volumes in case of tumor shrinkage during the course of radiochemotherapy. Hybrid devices combining a linear accelerator with magnetic resonance imaging (MRI-Linac) have recently been introduced into clinical practice. In addition to being able to use the superior soft tissue contrast of the MRI during treatment, the MRI-Linac also allows the treatment plan to be adapted daily to the anatomy of the day. These advantages allow the safety margins for rectal cancer dose escalation to be reduced to a few millimeters instead of 1 cm or more [2], [6]. At the same time, with such small margins accurate target volume definition becomes more crucial. Therefore, the goal of the present study was to evaluate the interobserver variability of MR based primary tumor delineation and to assess the impact of rectal ultrasound gel filling on this variability.

## Methods

Six patients with locally advanced rectal cancer of the mid or lower rectum were scanned in supine position on a 1.5 T MRI-Linac (1.5 T Unity MRI-Linac, Elekta, AB, Stockholm, Sweden) before start of their radiotherapy treatment. For the present interobserver agreement study, T2-weighted 3D pseudo steady-state sequences (TE = 168 ms, TR = 1300 ms) were used. Voxel size (mm^3^) was 1.2 × 1.2 × 1.2 mm^3^, the acquisition time 6:01 min (supplemental Table 1). All patients were scanned without and with 100 cc of ultrasound gel injected into the rectum as described previously [9]. Pseudonymized imaging datasets were subsequently distributed to eight radiation oncologists from seven centers. All participating radiation oncologists are affiliated with sites equipped with an MRI-Linac and have at least five years of experience in the treatment of gastrointestinal malignancies. The observers were instructed to delineate on the provided scans the gross tumor volume (GTV), defined as the gross visible extent of the rectal primary lesion. Relevant aspects from endoscopy reports were shared with the observers (supplemental document 2). First, only the scans without the ultrasound gel filling (MRI_e) were shared with the observers. Once the structure sets with the delineated tumor were returned to the coordinating center, the observers were provided with the MRI scans with the ultrasound gel filling (MRI_f). Again, the task was to delineate the primary tumor and return the structure set to the coordinating center.

To quantify the variability in tumor delineation two different approaches were used. For the first (Approach 1) a reference delineation was created by a radiation oncologist (CG) and a radiologist (SG). For this purpose, all available imaging data was considered, including diagnostic MRI scans, MRI_e, MRI_f and other daily MRI scans of patients treated on the MRI-Linac. The corresponding reference delineations were compared against each observer specific annotation with the quantitative Dice Similarity Index (DICE) and the 95 % Hausdorff distance (95 %HD), implemented in MATLAB R2020a (Mathworks Inc., Natick, MA, USA). For the second approach (Approach 2) all possible pairings between observers, detecting direct interobserver variability, were built and analyzed as previously reported [4] with DICE and 95 %HD determined in MATLAB. Paired T-tests were carried out to detect statistically significant differences between groups. A two-sided p < 0.05 was defined as statistically significant. All statistical tests were performed in GraphPad PRISM 9.1.2. Unless stated otherwise interquartile ranges (IQR) are reported with median values. The study was approved by the local ethics committee (659/2017BO1).

## Results

Five of the six patients were male with a median age of 59 years (range 39–73). All patients had stage T3 disease. All patients were able to complete the MRI with and without the rectal ultrasound gel filling.

**Fig. 1:**
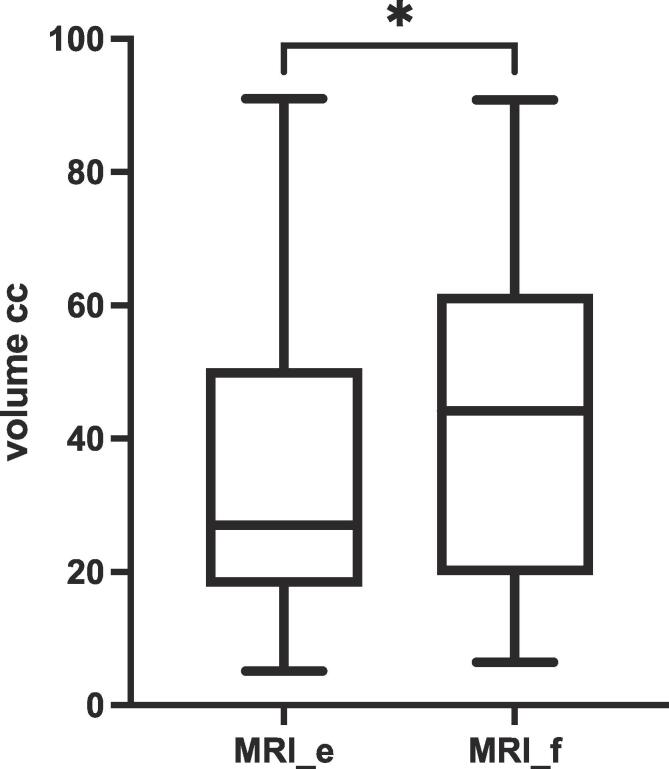
Box and whiskers plot of the delineated primary tumor volumes without (MRI_e) and with (MRI_f) previous application of rectal ultrasound gel.

**Fig. 2:**
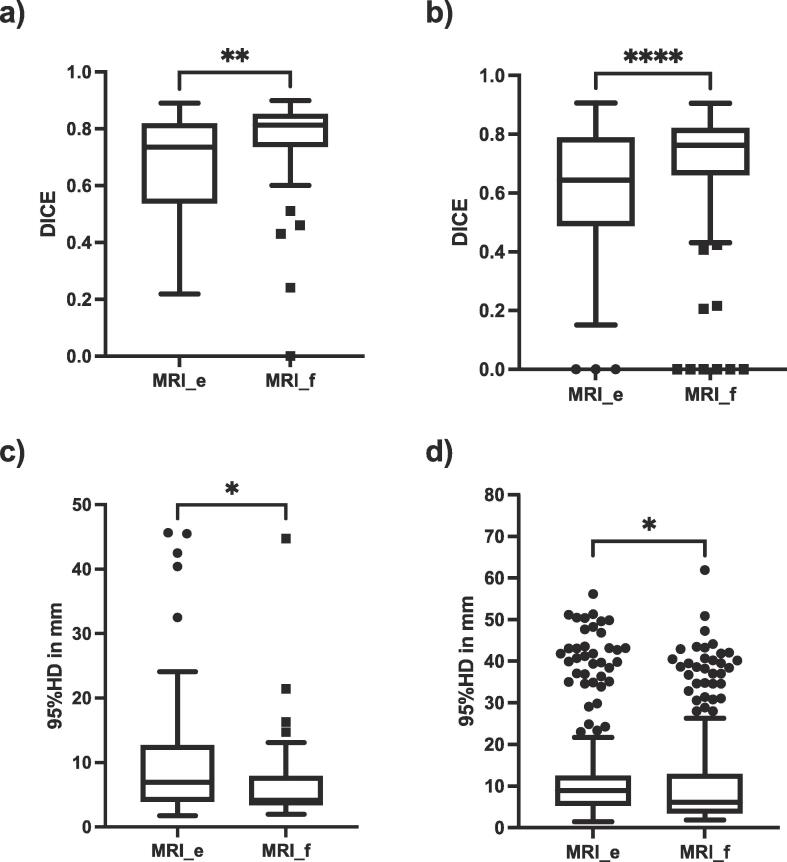
DICE coefficients (a and b) and 95% Hausdorff distances (c and d) for delineations of rectal primaries without (MRI_e) or with (MRI_f) rectal ultrasound filling. In a) and c) delineations were compared with a consensus delineation and in b) and d) all possible combinations between observers were evaluated.

**Supplementary Fig. 1:**
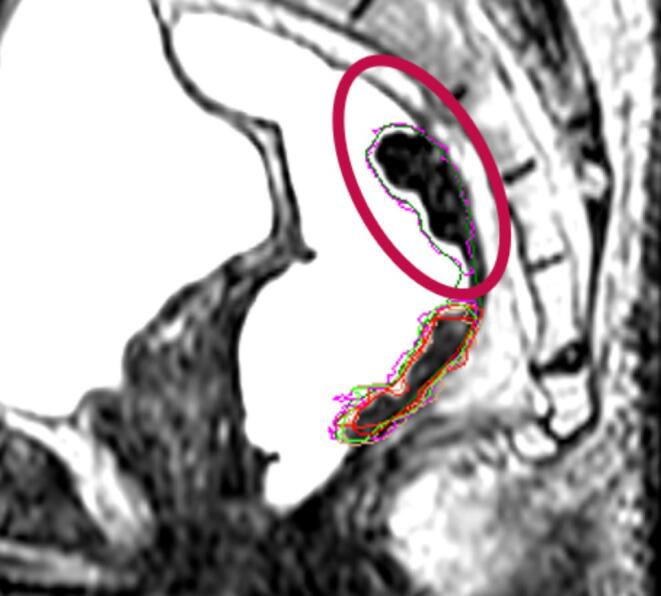
Feces adherent to the rectal wall in case 1 confused with actual tumor by one observer (red ellipse).

Delineated median [IQR] tumor volumes were 26.99 cc [18.01–50.34 cc] in MRI_e and 44.20 [19.72–61.59 cc] in MRI_f, p = 0.012, Fig. 1a. On an individual patient basis, the largest absolute difference was observed in case 4 with 42.62 cc [22.60–77.28 cc] in MRI_e and 68.25 cc [60.05–77.98 cc] in MRI_f (p = 0.04). The smaller median delineated volume in MRI_e was due to the omission of large parts of the tumor by four of the eight observers (supplemental Fig. 1).Fig. 2.

**Fig. 3:**
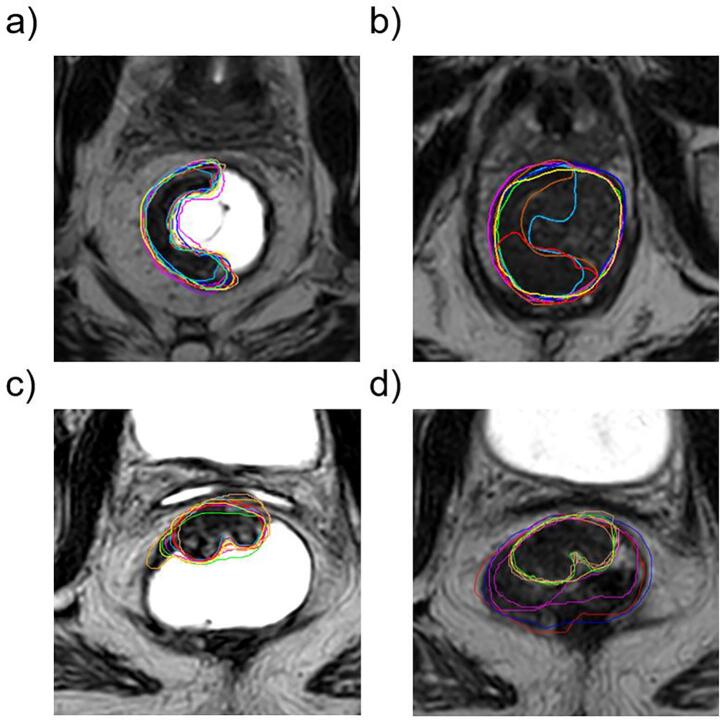
Representative examples of delineations returned by observers for case 2 (a) and b)) and case 6 (c) and d)). Sections a) and c) correspond to T2 MRI sequences with 100 cc of ultrasound gel applied prior to the scan and b) and d) to scans without the ultrasound gel.

Interobserver agreement using DICE was higher for MRI_f than for MRI_e. Median DICE for MRI_f was 0.81 [0.74–0.85] and 0.74 [0.54–0.82] for MRI_e using Approach 1 (p < 0.005) and 0.77 [0.66–0.82] for MRI_f vs 0.64 [0.49–0.79] for MRI_e for Approach 2, p < 0.0001. Median 95 %HD was significantly smaller in MRI_f relative to MRI_e (4.2 [3.5–7.8] mm vs 6.9 [4–12.6] mm, p = 0.04) for Approach 1 and 6.1 [3.6–13] vs mm. 8.9 [5.4–12] mm for Approach 2, p = 0.04). In one case a DICE of 0 was caused by the delineation of feces instead of the actual tumor (supplementary Fig. 2). Median values for DICE, 95 %HD and tumor volumes are summarized in Table 1. A representative example of delineations in MRI_e and MRI_f is shown in Fig. 3.

**Table 1:** Case by case summary of delineated tumor volumes, DICE coefficients and 95% Hausdorff distances.

	**Gender**	**Age**	**Volume**		**DICE (Approach 1)**		**DICE (Approach 2)**		**95 %HD (Approach 1)**		**95 %HD (Approach 2)**	
			MRI_e	MRI_f	MRI_e	MRI_f	MRI_e	MRI_f	MRI_e	MRI_f	MRI_e	MRI_f
**Case 1**	m	39	13 [9.4–22]	13 [9.8–17]	0.78 [0.54–0.82]	0.78 [0.68–0.81]	0.66 [0.49–0.79]	0.64 [0.46–0.72]	4.5 [2.2–6.3]	3.3 [2.6–3.9]	5.9 [3.2–12]	9.7 [2.9–39]
**Case 2**	m	63	41 [24–56]	49 [44–54]	0.74 [0.66–0.78]	0.83 [0.82–0.86]	0.63 [0.32–0.78]	0.81 [0.78–0.85]	8.5 [5.4–10]	2.7 [2.1–4]	9.4 [6.3–11]	4.1 [3.2–6.2]
**Case 3**	m	55	56 [45–72]	63 [52–72]	0.84 [0.74–0.87]	0.85 [0.83–0.87]	0.79 [0.71–0.84]	0.84 [0.8–0.86]	4.7 [3.5–8.7]	5.9 [5.3–7.8]	7.5 [5.4–11]	6.4 [4.9–9.1]
**Case 4**	m	62	43 [23–77]	68 [60–78]	0.67 [0.48–0.84]	0.83 [0.77–0.85]	0.5 [0.41–0.73]	0.77 [0.73–0.8]	24 [5.7–45]	8.9 [4.9–11]	9.5 [4.6–43]	7.7 [5.6–14]
**Case 5**	m	56	22 [14–37]	29 [22–61]	0.5 [0.41–0.63]	0.56 [0.44–0.77]	0.52 [0.43–0.64]	0.55 [0.46–0.68]	13 [11–16]	5 [3.3–9]	11 [7.6–17]	9.4 [3.3–25]
**Case 6**	f	73	24 [18–42]	20 [18–26]	0.75 [0.55–0.79]	0.78 [0.74–0.85]	0.66 [0.56–0.73]	0.76 [0.69–0.8]	6.7 [4.2–8.6]	3.6 [3.3–4]	8.1 [5.6–12]	5 [3.2–11]

## Discussion

This study investigated the interobserver variability in the delineation of rectal primary tumors based on MRIs acquired on a 1.5 Tesla MRI-Linac. Feasibility of the acquisition of MRI scans with 100 cc of rectal ultrasound gel filling in patients with rectal cancer on a 1.5 Tesla MRI-Linac was demonstrated. A higher agreement among observers was found when delineation was performed on MRI scans with the rectal application of 100 cc of ultrasound gel compared to without. There is a controversy about the benefits of rectal ultrasound filling in the diagnostic setting. This is especially due to the possibility that the rectal distention caused by the ultrasound filling might artificially shorten the distance of the tumor to the mesorectal fascia or increase the distance of the tumor from the dentate line [13], [14]. Moreover, the parts of the tumor that define risk feature such as infiltration depth or extramural venous invasion are extramural and not affected by higher contrast in the rectum.

These drawbacks are not relevant in the context of radiotherapy and there is in fact good rationale to use rectal ultrasound gel filling to facilitate more precise delineation of the primary tumor, particularly the intraluminal component. Accurate tumor delineation may be of limited relevance when standard doses of radiotherapy are applied and the primary tumor is treated with the adjacent rectal tissue, the mesorectum and lymphatics. However, if dose escalated radiotherapy is planned, precise definition of the tumor becomes crucially important. A dose response relationship for rectal tumors has previously been shown both in a *meta*-analysis and modeling studies [1], [3]. Dose escalation to achieve a clinical complete response and avoid radical surgery is particularly of interest in tumors with a low risk of distant spread. For high-risk tumors total neoadjuvant therapy (TNT) is now considered standard of care since it results in high rates of local tumor regression with a pathological complete response of almost 40 % and also significantly decreases distant metastases. While TNT is clearly an option for early tumors when organ preservation is intended, its benefits must be weighed against the toxicities of the intensified systemic treatment. With a non-adaptive cone beam CT based approach however, radiation dose escalation without an excess in side effects was not possible due to the large safety margins needed to account the variation from day to day in tumor position. For instance, Kleijnen et al. found that planning target margins of approximately 15 mm in craniocaudal extension and 10 mm in the anterior-posterior direction are required to ensure 95 % tumor coverage in 90 % of the cases [10]. The need for such large margins was likely a contributor to the negative results seen in the RECTAL-BOOST trial. In this trial patients were randomized to either radiochemotherapy with 50 Gy in 25 fractions over 5 weeks, or the same regimen with an upfront boost of 15 Gy in 5 daily fractions. Organ at risk constraints were prioritized over target volume coverage resulting in underdosage of the tumor boost volume in most cases, with a median dose more than 10 % less than the aimed dose [5]. With the advent of MRI-Linac hybrid devices it has become possible to considerably reduce planning target volume margins as treatment plans can be adapted to the anatomy of the day based on daily MRI scans. Several studies have shown that planning target volumes required to cover the remaining intrafractional motion with or without rectal filling can be reduced to 4 mm using an online adaptive workflow [2], [6], [9]. The present study demonstrates that the addition of rectal ultrasound gel results in more accurate target volume definition in rectal cancer. Another advantage from the rectal distension with gel filling is the distancing of uninvolved mucosa away from the tumor possibly leading to less toxicity [9].

With regards to study limitations, we acknowledge that in a real life setting potentially more information regarding the tumor, such as images from endoscopy would have been available in addition to the images provided to the observers. Unexpectedly the median GTV volumes were found to be higher in the MRI scans with endorectal filling in some cases. This was due to the circumstance that large parts of the GTVs have been missed by some observers in the MRI_e scans, consequence that in a theoretical clinical setting would have led to incomplete coverage of the target volume. This finding confirms our hypothesis that with the application of endorectal gel filling the primary rectal tumor volume can be better identified, crucial aspect in the context of radiation dose escalation.

We further acknowledge that there is no perfect “gold standard” for delineation and that another limit of the present study is the small sample size. Finally, only baseline scans were used for the purpose of this delineation study, however we believe that the findings of the study can be extrapolated to MRIs acquired during radiotherapy in the context of a weekly adaptive dose escalation protocol.

## Conclusion

In the present study we show that the application of ultrasound gel filling decreases interobserver variability in the delineation of rectal primary tumors and might become an effective tool for use in future dose escalation protocols.

## Declaration of Competing Interest

The authors declare the following financial interests/personal relationships which may be considered as potential competing interests: The Department of Radiation Oncology Tübingen (SB, CG, DT, DZ) receives within the frame of research agreements financial and technical support as well as sponsoring for travels and scientific symposia from: Elekta AB (Stockholm, Sweden), Philips GmbH, Siemens, PTW Freiburg Physikalisch-Technische Werkstätten Dr. Pychlau GmbH. SB: Honoraria for talks by Elekta. AT: Travel and speaking funding by Elekta. from Elekta. PBR is supported by an NIH/NCI grant (K08CA255574), the Memorial Sloan Kettering Cancer Center, an NIH Loan Repayment Program (LRP) award. PBR and RS are supported in part by a National Institutes of Health/National Cancer Institute (NIH/NCI) Memorial Sloan Kettering Cancer Center (MSK) Support Grant (P30 CA008748).

## Appendix A Supplementary data

Supplementary Table 1 Magnetic resonance imaging parameters.

Supplementary Table 2 Information from endoscopy reports available to the observers.
