# Supplementary material for: Impact of endorectal filling on interobserver variability of MRI based rectal primary tumor delineation
Source: Clin Transl Radiat Oncol. 2022 Sep 21;38:1–5. doi: 10.1016/j.ctro.2022.09.002 (PMC9589000; doi:10.1016/j.ctro.2022.09.002)
Supplement: Supplementary Table 1 — Magnetic resonance imaging parameters. [file mmc1.docx]

| **Parameter** | **T2-weighted**  **3D pseudo steady-state**  (**T2w-6min**) |
| --- | --- |
| Matrix size | 332 × 371 |
| FOV (mm^3^) | 400 × 448 × 270 |
| Voxel (mm^3^) | 1.2 × 1.2 × 1.2 |
| TE (ms) | 168 |
| TR (ms) | 1300 |
| Flip angle (°) | 90 |
| WFS(pix) / BW(Hz) | 0.519 / 418.3 |
| Acquisition time (min) | 6:01 |

FOV, field of view; TE, echo time; TR, repetition time; WFS, water-fat shift; BW, bandwidth
