# Supplementary material for: Impact of endorectal filling on interobserver variability of MRI based rectal primary tumor delineation
Source: Clin Transl Radiat Oncol. 2022 Sep 21;38:1–5. doi: 10.1016/j.ctro.2022.09.002 (PMC9589000; doi:10.1016/j.ctro.2022.09.002)
Supplement: Supplementary Table 2 — Information from endoscopy reports available to the observers. [file mmc2.docx]

| **Case** | **Endoscopy findings** |
| --- | --- |
| 1 | On rectoscopy distal end of the tumor located dorsally, 4cm from dentate line, dorsally extending from 5-7 o'clock position |
| 2 | Supraanal rectal tumor, semicircularly extending from 6 to 11 o'clock, 0 cm from dentate line, cannot be passed with rectoscope  *NB: The patient has a presacral mass on MRI, it was scored as benign, please “ignore” this for the current delineation study.* |
| 3 | Palpable with finger tip, tumor covers two thirds of the circumference, distal end: 4 cm from anal verge, proximal end 10 cm |
| 4 | Circular stenosing tumor, proximal end at 10 cm from anal verge |
| 5 | Tumor with a longitudinal extension of approximately 3cm, distal end approx. 6cm from anal verge |
| 6 | Tumor palpable on DRU, on endoscopy begins approximately 2-3 cm above the sphincter. Can easily be passed with the endoscope. |
